# Clinical features and prognostic factors analysis of intravenous leiomyomatosis

**DOI:** 10.3389/fsurg.2022.1020004

**Published:** 2023-01-30

**Authors:** Jingying Chen, Hualei Bu, Zhaoyang Zhang, Ran Chu, Gonghua Qi, Chen Zhao, Qiuman Wang, Xinyue Ma, Huan Wu, Zhiyuan Dou, Xia Wang, Beihua Kong

**Affiliations:** ^1^ Department of Obstetrics and Gynecology, Qilu Hospital of Shandong University, Jinan, China; ^2^ Gynecologic Oncology Key Laboratory of Shandong Province, Qilu Hospital of Shandong University, Jinan, China

**Keywords:** intravenous leiomyomatosis, prognostic factor, treatment, surgery, progression-free survival

## Abstract

**Background:**

The treatment and prognostic factors of intravenous leiomyomatosis (IVL) remain lacking systematic evidence.

**Methods:**

A retrospective study was conducted on IVL patients from the Qilu Hospital of Shandong University, and IVL cases were published in PubMed, MEDLINE, Embase and Cochrane Library databases. Descriptive statistics were used for the basic characteristics of patients. The Cox proportional hazards regression analysis was used to assess the high-risk factors related to the progression-free survival (PFS). The comparison of survival curves was performed by Kaplan–Meier analysis.

**Results:**

A total of 361 IVL patients were included in this study, 38 patients from Qilu Hospital of Shandong University, and 323 patients from the published literature. Age ≤45 years was observed in 173 (47.9%) patients. According to the clinical staging criteria, stage I/II was observed in 125 (34.6%) patients, and stage III/IV was observed in 221 (61.2%) patients. Dyspnea, orthopnea, and cough were observed in 108 (29.9%) patients. Completed tumor resection was observed in 216 (59.8%) patients, and uncompleted tumor resection was observed in 58 (16.1%) patients. Median follow-up period was 12 months (range 0–194 months), and 68 (18.8%) recurrences or deaths were identified. The adjusted multivariable Cox proportional hazard analysis showed age ≤45 years (vs. >45) (hazard ratio [HR] = 2.09, 95% confidence interval [CI] 1.15–3.80, *p* = 0.016), and uncompleted tumor resection (vs. completed tumor resection) (HR = 22.03, 95% CI 8.31–58.36, *p* < 0.001) were high-risk factors related to the PFS.

**Conclusion:**

Patients with IVL have a high probability of recurrence after surgery and a poor prognosis. Patients younger than 45 years and with uncompleted tumor resection are at higher risk of postoperative recurrence or death.

## Introduction

Intravascular leiomyomatosis (IVL) is a histologically-benign, rare mesenchymal tumor with a biological behavior similar to that of a malignant tumor. It is characterized by the growth of a benign leiomyoma along the intra- and extrauterine venous lumen forming a tumor thrombus that can extend to the inferior vena cava, right atrium, right ventricle, and pulmonary artery, and cause sudden death in severe cases (1, 2).

The incidence of IVL is low, with approximately 400 cases reported in the literature since it was first documented in 1896 (3). Clinical manifestations in early-stage patients are non-specific, with approximately 30% of the patients showing no symptoms or specific tumor markers, and the postoperative recurrence rate is approximately 14.0 − 27.8% (4, 5). In recent years, the emergence of imaging technologies such as computed tomography (CT), CT angiography, and magnetic resonance imaging (MRI) has provided an invaluable tool for the diagnosis of intravenous tumors and the evaluation of the scope of tumor involvement, improving the accuracy of surgical evaluation and the success rate of surgery (6, 7). However, the number of IVL cases remains underestimated due to its relatively obscure onset, and as current treatment is mainly derived from case reports, there is a lack of solid evidence to guide clinical treatment.

Therefore, we have conducted this retrospective study and systematic review to analyze the epidemiology, history, pathology, diagnosis, treatment, and prognosis of IVL, and the risk factors affecting the prognosis of patients were also analyzed.

## Materials and methods

### General information

**Figure 1:**
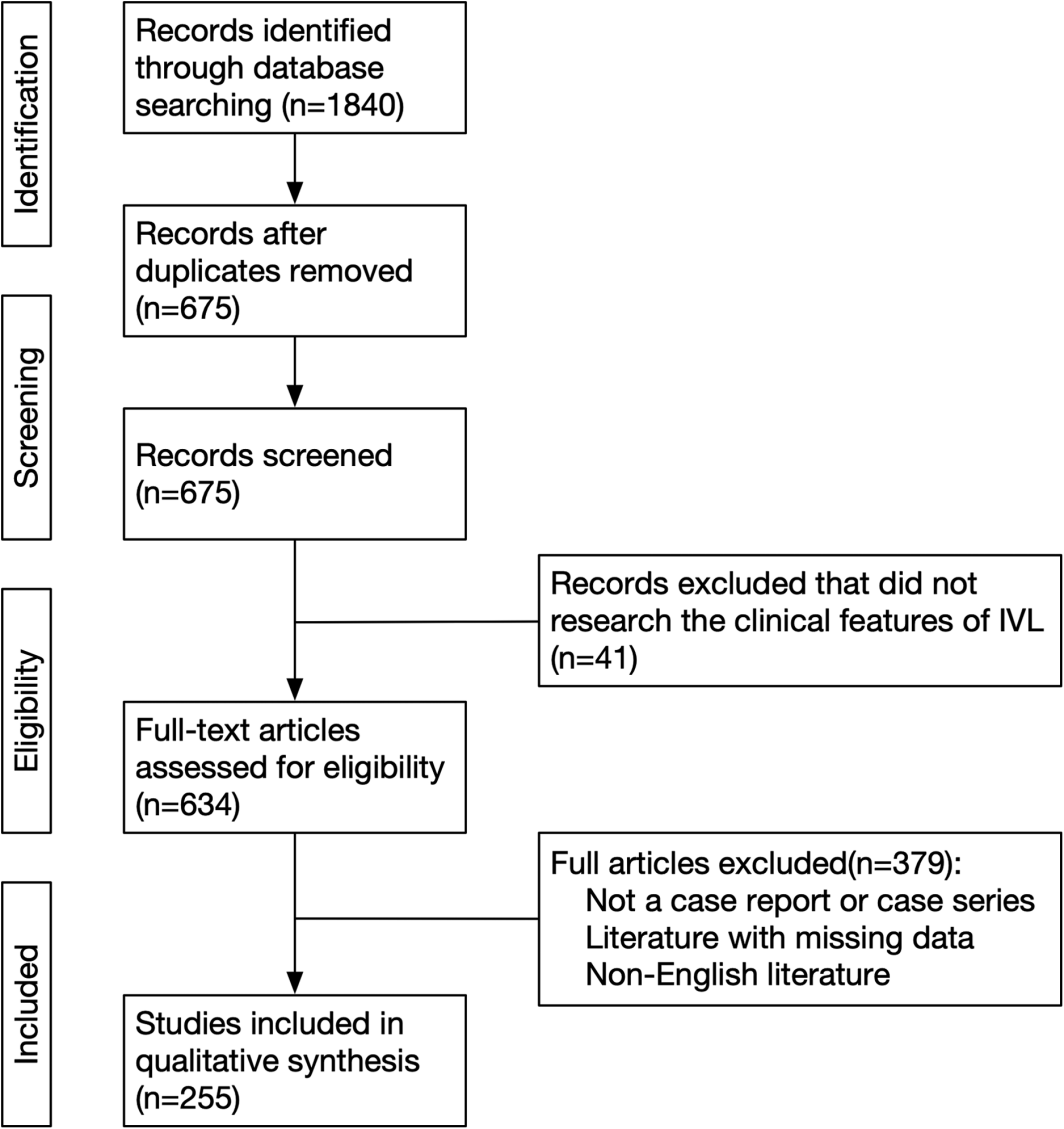
Flow diagram of literatures searching. IVL, intravenous leiomyomatosis.

323 IVL patients in 255 studies were included in our study (Figure 1) (2, 7–260). Patients diagnosed with IVL in Qilu Hospital of Shandong University from January 1, 2007, to December 31, 2021 were enrolled in our study. The follow-up period ended on March 31, 2022. The PubMed, MEDLINE, Embase, and Cochrane Library databases were searched using the keywords “intravenous leiomyomatosis,” “intracardiac leiomyomatosis,” and “IVL.” The search period covered from January 1, 1959, to March 31, 2021. Of 1,840 studies screened, 323 IVL patients in 259 studies were included in our study (Figure 1) (8–242). Literature inclusion criteria were as follows: article type was a case report or case series, and the language was English; exclusion criteria included: irrelevant literature, duplicate case reports published by the same hospital or the same author, and literature with missing data. The following clinical characteristics were collected: age, gravidity, parity, history of hysteromyoma, clinical staging, tumor size, operation and pathological details, immunohistochemical characteristics [such as Ki-67, smooth muscle actin (SMA), desmin, estrogen receptor (ER), progesterone receptor (PR), and CD34], and follow-up time.

### Clinical staging of IVL

Patients were classified as stage I−IV based on the clinical staging system proposed by Ma et al., which reflects the preoperative progression of the tumor (261). Stage I referred to tumors that had penetrated the uterine venous wall but were confined to the pelvic cavity; stage II referred to tumors that had extended into the abdominal cavity but had not reached the renal vein; stage III referred to tumors that had reached the renal vein and inferior vena cava and had further extended into the right atrium, but had not reached the pulmonary arteries; and finally, and stage IV referred to tumors that had reached the pulmonary arteries and/or lung metastases were observed.

### Endpoints

The primary endpoint of this study was the progression-free survival (PFS). PFS is described as the time interval from primary treatment to the first evidence of tumor recurrence, death, or last follow-up.

### Statistical analysis

Descriptive statistics were used to describe the basic characteristics, the grouped data was described as *n* (%), and the continuous data was described as the median (range). Univariable and adjusted multivariable Cox proportional hazards regression analysis were used to identify the high-risk factor affecting PFS, and results were described as hazard ratios (HR), associated 95% confidence intervals (CI), and *p*-values. The Kaplan-Meier method with the log-rank test was conducted to evaluate the effects of high-risk factors on PFS. Statistical analysis was conducted with SPSS (version 26.0), The *p* value <0.05 is considered significant.

## Results

Patient characteristics are shown in Table 1. A total of 361 IVL patients were included in this study, 323 from the literature, and 38 from the Qilu Hospital of Shandong University. Age ≤45 years was observed in 173 (47.9%) patients. Dyspnea, orthopnea, and cough were observed in 108 (29.9%) patients. Completed tumor resection was observed in 216 (59.8%) patients, and uncompleted tumor resection was observed in 58 (16.1%) patients. According to the clinical staging criteria, stage I/II was observed in 125 (34.6%) patients, and stage III/IV was observed in 221 (61.2%) patients. The Median follow-up period was 12 months (range 0–194 months), and 68 (18.8%) recurrences or deaths were identified. The global regional distribution of all enrolled patients is shown in Supplementary Figure S1.

**Table 1:** Clinical characteristics of IVL patients.

Characteristics	Total (*n* = 361)	Patients from Qilu hospital (*n* = 38)	Patients from literature review (*n* = 323)
Age (years)			
≤45	173 (47.9)	24 (63.2)	149 (46.1)
>45	184 (51.0)	14 (36.8)	170 (52.6)
Unknown	4 (1.1)	0 (0.0)	4 (1.2)
Gravidity			
≤2	70 (19.4)	18 (47.4)	52 (16.1)
>2	51 (14.1)	20 (52.6)	31 (9.6)
Unknown	240 (66.5)	0 (0.0)	240 (74.3)
Parity			
≤1	61 (16.9)	26 (68.4)	35 (10.8)
>1	70 (19.4)	12 (31.6)	58 (18.0)
Unknown	230 (63.7)	0 (0.0)	230 (71.2)
History of hysteromyoma			
Yes	149 (41.3)	35 (92.1)	114 (35.3)
No	7 (1.9)	2 (5.3)	5 (1.5)
Unknown	205 (56.8)	1 (2.6)	204 (63.2)
Clinical staging			
I/II	125 (34.6)	29 (76.3)	96 (29.7)
III/IV	221(61.2)	9 (23.7)	212 (65.6)
Unknown	15 (4.2)	0 (0.0)	15 (4.6)
Length of the tumor in vessel (cm)			
≤15	102 (28.2)	28 (73.7)	74 (22.9)
>15	92 (25.5)	7 (18.4)	85 (26.3)
Unknown	167 (46.3)	3 (7.9)	164 (50.8)
Clinical symptom			
Dyspnea / orthopnea / cough	108 (29.9)	3 (7.9)	105 (32.5)
Menstrual changes	57 (15.8)	5 (13.2)	52 (16.1)
Lower limb swelling or pain	62 (17.2)	2 (5.3)	60 (18.6)
Abdominal pain / distention / discomfort	87 (24.1)	14 (36.8)	73 (22.6)
Dizziness / syncope	55 (15.2)	2 (5.3)	53 (16.4)
Palpitation / tachycardia	39 (10.8)	0 (0.0)	39 (12.1)
Thoracalgia / chest tightness	36 (10.0)	1 (2.6)	35 (10.8)
Hypodynamia	15 (4.2)	0 (0.0)	15 (4.6)
Urinary frequency / incontinence / dysuria	10 (2.8)	4 (10.5)	6 (1.9)
Others	9 (2.5)	0 (0.0)	9 (2.8)
None	100 (27.7)	15 (39.5)	85 (26.3)
Hysterectomy			
Yes	292 (80.9)	30 (78.9)	262 (81.1)
No	60 (16.6)	8 (21.1)	52 (16.1)
Unknown	9 (2.5)	0 (0.0)	9 (2.8)
Oopherectomy			
Yes	167 (46.3)	20 (52.6)	147 (45.5)
No	155 (42.9)	18 (47.4)	137 (42.4)
Unknown	39 (10.8)	0 (0.0)	39 (12.1)
Completed tumor resection			
Yes	216 (59.8)	35 (92.1)	181 (56.0)
No	58 (16.1)	3 (7.9)	55 (17.0)
Unknown	87 (24.1)	0 (0.0)	87 (26.9)
Surgical approach			
One-stage	55 (15.2)	2 (5.3)	53 (16.4)
Two-stage	38 (10.5)	2 (5.3)	36 (11.1)
Others	268 (74.2)	34 (89.5)	234 (72.4)
Follow-up time (months)	12 (0-194)	33 (0-178)	9 (0-194)
Recurrence/death	68 (18.8)	7 (18.4)	61 (18.9)

Value are *n* (%) or median (range). One patient usually has multiple clinical manifestations, so the percentage of symptoms in all patients is more than 100%. IVL, intravenous leiomyomatosis.

### IVL patients from Qilu Hospital of Shandong University

Clinical characteristics of 38 IVL patients from Qilu Hospital of Shandong University are shown in Supplementary Table S1. From January 1, 2007, to December 31, 2021, A total of 31,594 patients with uterine fibroid were admitted to the Qilu Hospital of Shandong University, and 38 (1.2‰) IVL patients were diagnosed. The median age of the 38 IVL patients was 43 years (range 33−66). Abdominal discomfort (36.8%) was the most common clinical manifestation, followed by menstrual abnormalities (13.2%), urinary (10.5%), and cardiovascular symptoms (7.9%), which were mainly related to the site of the lesion involvement. In terms of clinical staging, stage I/II was observed in 29 (76.3%) patients, and stage III/IV was observed in 9 (23.7%) patients. All visible tumors were removed in 35 (92.1%) patients. One patient with stage IV disease died during surgery due to intraoperative left pulmonary artery hemorrhage. After a median follow-up period of 33 months (range 0–178 months), 6 recurrences and 1 death were identified.

### IVL patients from literature

The median age at diagnosis was 46 years (range 21−81). Among the patients for whom clinical data could be obtained, 149 (46.1%) had a parturition number exceeded once. Stage I/II was observed in 96 (29.7%) patients, and stage III/IV was observed in 212 (65.6%) patients. The main clinical symptoms were respiratory discomfort (32.5%), and other common symptoms included abdominal discomfort (22.6%), lower limb edema (18.6%), abnormal menstruation (16.1%), and dizziness (16.4%). Remarkably, 26.3% of patients showed no symptoms. Among the patients who underwent surgical treatment, 181 patients (56.0%) had completed tumor resection. After a median follow-up period of 9 months (range 0–194 months), 61 recurrences and 11 deaths were identified (Supplementary Table S2).

### Immunohistochemical characteristics of IVL patients

Immunohistochemical results are summarized in Table 2. A total of 19 patients underwent Ki-67 immunohistochemical analysis, with a positivity rate of less than 5% in 17 patients. Immunohistochemical analysis of SMA and desmin was performed in 45 and 41 patients, respectively, with a positivity rate of 100%. In addition, the positivity rate for the presence of ER was 97.4% (38/39), PR was 91.7% (33/36), and CD34 was 69.2% (9/13), respectively.

**Table 2:** Immunohistochemical characteristics of IVL patients.

Immunohistochemical characteristics	Total	Patients from Qilu hospital	Patients from literature review
Ki-67			
Positive rate ≤5%	17	7 (100.0)	10 (83.3)
Positive rate >5%	2	0 (0.0)	2 (16.7)
SMA			
Positive	45	9 (100.0)	36 (100.0)
Negative	0	0 (0.0)	0 (0.0)
Desmin			
Positive	41	9 (100.0)	32 (100.0)
Negative	0	0 (0.0)	0 (0.0)
ER			
Positive	38	4 (100.0)	34 (97.1)
Negative	1	0 (0.0)	1 (2.9)
PR			
Positive	33	3 (75.0)	30 (93.8)
Negative	3	1 (25.0)	2 (6.2)
CD34			
Positive	9	5 (83.3)	4 (57.1)
Negative	4	1 (1.7)	3 (2.9)

Value are *n* (%). IVL, intravenous leiomyomatosis; SMA, smooth muscle actin; ER, estrogen receptor; PR, progesterone receptor.

### High-risk factor analysis of PFS

**Figure 2:**
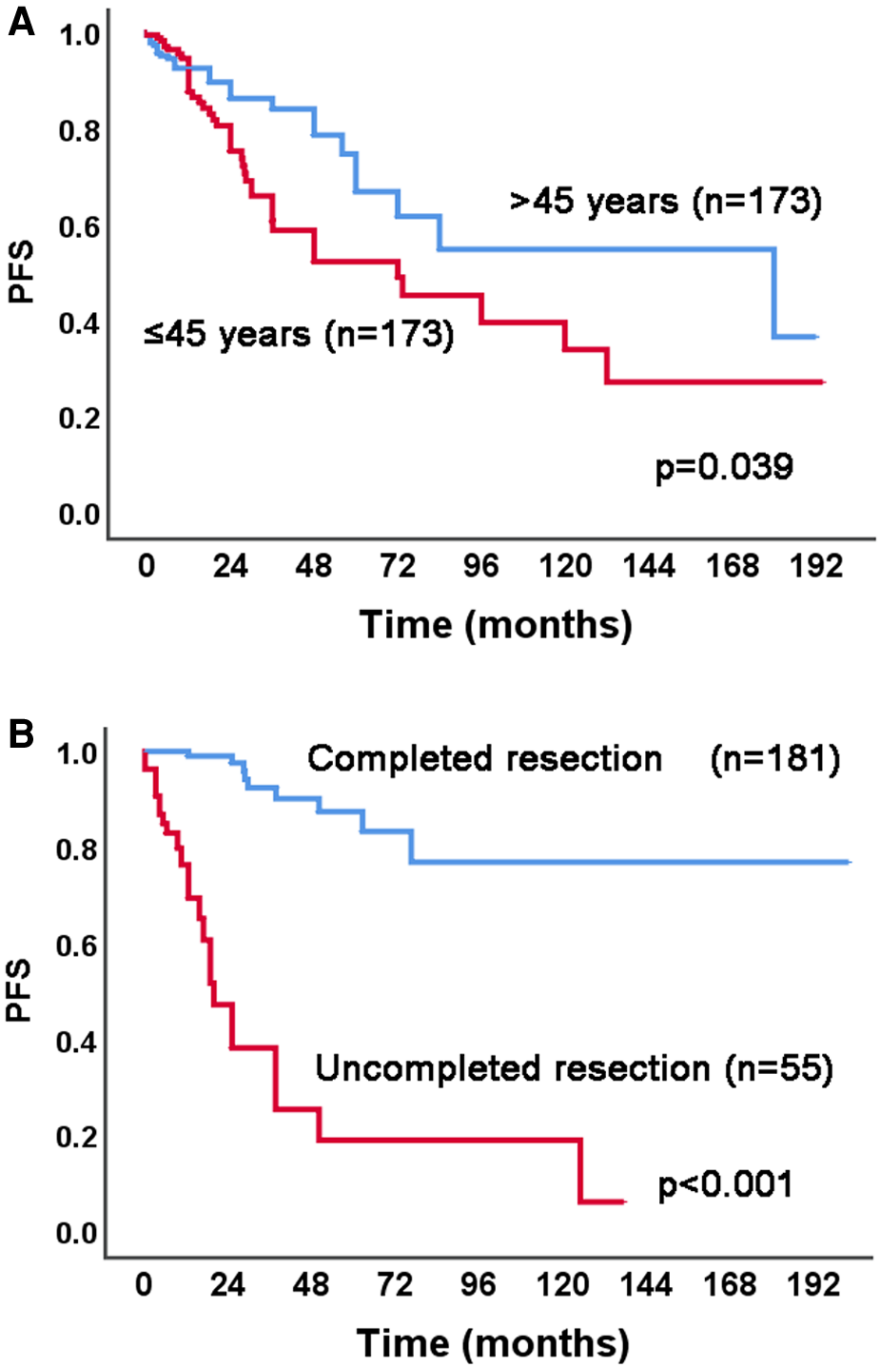
Kaplan–Meier analysis of high-risk factors for PFS in 361 IVL patients. PFS, progression-free survival; IVL, intravenous leiomyomatosis.

**Figure 3:**
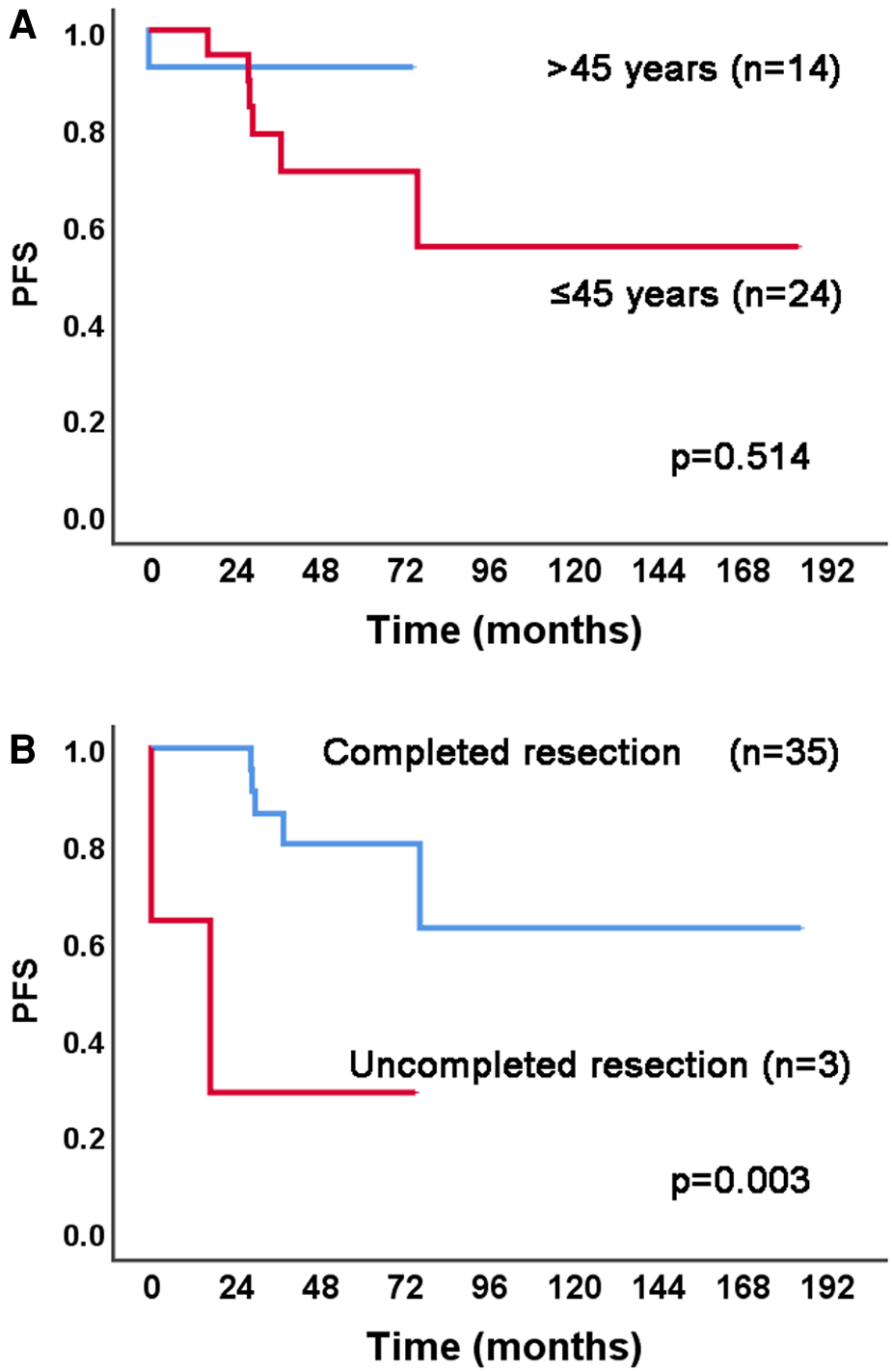
Kaplan–Meier analysis of high-risk factors for PFS in 38 IVL patients from Qilu hospital of shandong university. PFS, progression-free survival; IVL, intravenous leiomyomatosis.

The results of univariable and adjusted multivariable Cox proportional hazards regression analysis for PFS are shown in Table 3. After the adjusted multivariable Cox proportional hazard analysis, age ≤45 years (vs. > 45) (HR = 2.09, 95% CI 1.15–3.80, *p* = 0.016), and uncompleted tumor resection (vs. completed tumor resection) (HR = 22.03, 95% CI 8.31–58.36, *p* < 0.001) were selected as high-risk factors related to the PFS. Figure 2 shows the Kaplan–Meier curves of the high-risk factors for PFS in all 361 IVL patients, and Figure 3 shows the Kaplan–Meier curves of the high-risk factors for PFS in 38 IVL patients from Qilu Hospital of Shandong University.

**Table 3:** Univariable and adjusted multivariable Cox proportional hazards regression analysis for PFS of 361 IVL patients.

	Univariable analysis		Adjusted multivariable analysis	
Characteristics	HR (95%CI)	*p* value	HR (95%CI)	*p* value
Age (years)		0.129		0.054
≤45	1.67 (1.02–2.73)	0.043	2.09 (1.15–3.80)	0.016
>45	Reference		Reference	
Unknown	–	–	–	–
Gravidity		0.543		0.068
≤2	Reference		Reference	
>2	1.26 (0.54–2.92)	0.596	2.88 (1.08–7.71)	0.035
Unknown	1.43 (0.76–2.70)	0.273	0.60 (0.07–5.28)	0.644
Parity		0.388		0.845
≤1	Reference		Reference	
>1	1.34 (0.59–3.04)	0.486	0.81 (0.31–2.11)	0.670
Unknown	1.58 (0.82–3.07)	0.175	1.27 (0.13–12.17)	0.837
History of hysteromyoma		0.899		0.664
Yes	0.75 (0.18–3.19)	0.699	0.56 (0.11–2.86)	0.481
No	Reference		Reference	
Unknown	0.82 (0.20–3.40)	0.783	0.49 (0.10–2.42)	0.384
Clinical staging		0.100		0.092
I/II	Reference		Reference	
III/IV	1.39 (0.81–2.39)	0.228	1.08 (0.48–2.42)	0.859
Unknown	–	–	–	–
Length of the tumor in vessel (cm)		<0.001		0.082
≤15	Reference		Reference	
>15	1.04 (0.39–2.74)	0.939	0.63 (0.23–1.75)	0.377
Unknown	3.76 (1.90–7.43)	<0.001	1.66 (0.74–3.74)	0.222
Hysterectomy		0.006		0.066
Yes	0.55 (0.31–0.95)	0.032	0.68 (0.34–1.36)	0.275
No	Reference		Reference	
Unknown	2.65 (0.77–9.16)	0.124	4.91 (0.93–25.86)	0.060
Oopherectomy		0.016		0.132
Yes	Reference		Reference	
No	2.09 (1.22–3.57)	0.007	1.09 (0.55–2.18)	0.805
Unknown	2.45 (1.07–5.62)	0.034	0.34 (0.10–1.23)	0.099
Completed tumor resection		<0.001		<0.001
Yes	Reference		Reference	
No	14.69 (6.80–31.75)	<0.001	22.03 (8.31–58.36)	<0.001
Unknown	9.66 (4.64–20.10)	<0.001	21.62 (8.19–57.09)	<0.001
Surgical approach		0.543		0.129
One-stage	Reference		Reference	
Two-stage	0.90 (0.29–2.84)	0.858	0.73 (0.21–2.61)	0.633
Others	1.36 (0.62–3.01)	0.447	0.39 (0.15–1.01)	0.052

PFS, progression-free survival; IVL, intravenous leiomyomatosis; HR, hazard ratio; CI, confidence interval.

## Discussion

The diagnosis and treatment of IVL have received increasing attention from clinicians. However, the available studies are still in the form of case reports and lack a cohort analysis. Our study included a total of 361 IVL patients, the clinical characteristics and pathological findings were described in detail. And the multivariable Cox proportional hazard regression analysis showed patients younger than 45 years and with uncompleted tumor resection were high-risk factors for PFS.

In this study, 27.7% of IVL patients showed no symptoms or signs. Common signs included pelvic pain, abdominal discomfort, and menorrhagia. In advanced-stage cases, when the inferior vena cava or the right heart and pulmonary artery were involved, some patients had dyspnea, chest pain, lower limb edema, and syncope, while some patients had no cardiopulmonary symptoms before the occurrence of cardiac insufficiency, pulmonary embolism, or sudden death (7, 13, 69, 244). Therefore, IVL has an insidious onset and complicated clinical manifestations, and lacks specificity, with patients usually noticing IVL only after the tumor has invaded the inferior vena cava or above, causing serious clinical symptoms that are difficult to diagnose.

In 2016, Ma et al. first proposed a clinical staging system that reflects the preoperative progression of tumors (261). Among the IVL patients admitted to the Qilu Hospital of Shandong University, stage III and IV patients only accounted for 23.7%, whereas in all the collected cases, they accounted for as high as 61.2% of the total number of patients. However, this did not represent the proportion of patients with IVL involving the inferior vena cava and cardiopulmonary system, as there might be many asymptomatic IVL patients; moreover, some early IVL patients might have been undiagnosed, or some early cases with relatively limited lesions might have been unreported due to the limitations of the medical treatment. Patients in stages III and IV have a higher risk of sudden or surgical deaths due to the possible tumor adhesion to the vascular wall, internal structure of the heart, or complete outflow tract obstruction (4, 13, 36). Eleven IVL-related deaths occurred in stage III and IV of all included cases, while no deaths occurred in stage I and II patients.

The exact pathogenesis of IVL is still unclear, and there are several theories about its origin. One theory held that IVL originated from the vessel wall (1); another theory postulated that IVL was the result of uterine leiomyoma invading the vascular muscle layer (262, 263); other researchers proposed that IVL might originate from the myometrium rather than fibroids (9, 238, 264). Kir et al. performed immunohistochemical ER and PR detection in tumor tissues and adjacent vascular walls of IVL patients, respectively, and the results showed that ER and PR were positively expressed to varying degrees (10%–60% and 10%–70%, respectively). However, ER and PR were absent or weakly expressed in the vascular endothelial and subendothelial cells. This confirmed that IVL originated from the uterus and not the blood vessel wall (262). In this study, immunohistochemical results were summarized, and the positive rates of ER and PR were 97.4% and 91.7%, respectively, confirming the hypothesis that IVL originates from the uterus to a certain extent. In addition, immunohistochemical analysis showed that the SMA and desmin positive rates were both 100%, suggesting that IVL has molecular cytogenetic characteristics similar to those of uterine leiomyoma (265). Ki-67 is widely used as a potential prognostic marker in the study of malignant diseases as it represents the degree of cell proliferation and is proportional to the degree of tumor malignancy (266, 267). Among the included IVL cases, 89.5% of patients had a Ki-67 positive rate of less than 5%. This indicated that although IVL shares certain biological behaviors with malignant tumors, the degree of malignancy was very low, and its mortality mainly resulted from complications caused by the cardiopulmonary metastasis of the tumors.

Surgical resection is the main treatment for IVL; however, there is still a lack of corresponding guidelines, and the current treatment is mainly based on limited case reports. Currently, the recommended surgical methods are radical tumor resection, including hysterectomy, bilateral salpingo-oophorectomy, and extrauterine tumor resection to remove all visible tumor and reduce the risk of future recurrence. Completed resection of all visible tumor can reduce the risk of recurrence, and several studies have reached this consistent conclusion (261, 268, 269). However, ovarian resection is still very cautiously applied, as the impact of oophorectomy on the endocrine function of women is crucial. As IVL is associated with high estrogen expression, it would be intuitive to assume that ovarian preservation is not recommended, and several studies have suggested bilateral oophorectomy to reduce recurrence (53, 261, 270); however, there is still insufficient evidence to confirm this conclusion. Peng J. et al. repoted a retrospective study of 166 IVL patients who accepted surgery treatment, similar to our findings, compared with total hysterectomy and bilateral salpingo-oophorectomy, total hysterectomy does not increase the risk of recurrence (odds ratio = 0.96, 95% CI 0.08–10.58, *p* = 0.96), but tumorectomy seems a high-risk factor of recurrence (odds ratio = 20.09, 95% CI 4.16–97.10, *p* < 0.01) (271).

Two surgical procedures were mainly performed in patients with lesions involving the cardiovascular system: one-stage surgery was a combined exploration of the chest, abdomen, and pelvis, with resection of the lesions in the pelvic and abdominal cavity and tumors involving the cardiovascular system, but conversely, two-stage surgery referred to the removal of lesions involving the cardiovascular system, followed by secondary surgery to remove the tumors in the abdominal and pelvic cavities. In a small number of cases, tumor resection of the cardiovascular system was performed only due to the patient's refusal to further surgery or the impossibility of removing the pelvic and intraperitoneal tumors. The two surgical procedures have advantages and disadvantages: extracorporeal circulation in one-stage surgery can effectively control intraoperative bleeding, protect vital organs, and provide better exposure to the surgical field and sufficient time for tumor resection. In addition, it can reduce the possibility of intraoperative embolism. However, one-stage surgery requires high anesthesia techniques and long operation times and increases the risk of coagulation dysfunction caused by heparin and the incidence of intraoperative and postoperative complications. Two-stage surgery avoids the shortcomings of one-stage surgery, but there is a risk of pulmonary embolism caused by postoperative residual tumor embolism, which increases pain and hospitalization costs (272). Furthermore, our research confirmed that there was no difference between one-stage and two-stage surgeries in the prognosis of the patients, and appropriate surgical methods could be selected according to their conditions.

Still, due to the limitations inherent to a retrospective study, our research had some limitations. First, the publication year of the literature included in the study was relatively early, and there was inevitably some missing information. Second, although several published articles have been included, the number of patients in our study was relatively small. In future studies, increasing the sample size may compensate for the above research limitations.

## Conclusion

The clinical manifestations of patients with IVL are not typical, especially in the early stages of the disease, and they are mainly related to the site of tumor involvement. The postoperative recurrence and death incidence was higher in young and uncomplete tumor resection patients.

## Data Availability

The original contributions presented in the study are included in the article/**Supplementary Material**, further inquiries can be directed to the corresponding author/s.
