# Supplementary material for: Clinical features and prognostic factors analysis of intravenous leiomyomatosis
Source: Front Surg. 2023 Jan 30;9:1020004. doi: 10.3389/fsurg.2022.1020004 (PMC9922872; doi:10.3389/fsurg.2022.1020004)
Supplement: Supplementary file 1 [file Datasheet1.pdf]

Supplemental Material

The supplemental material includes:

- **Figure S1**
- **Table S1**
- **Table S2**

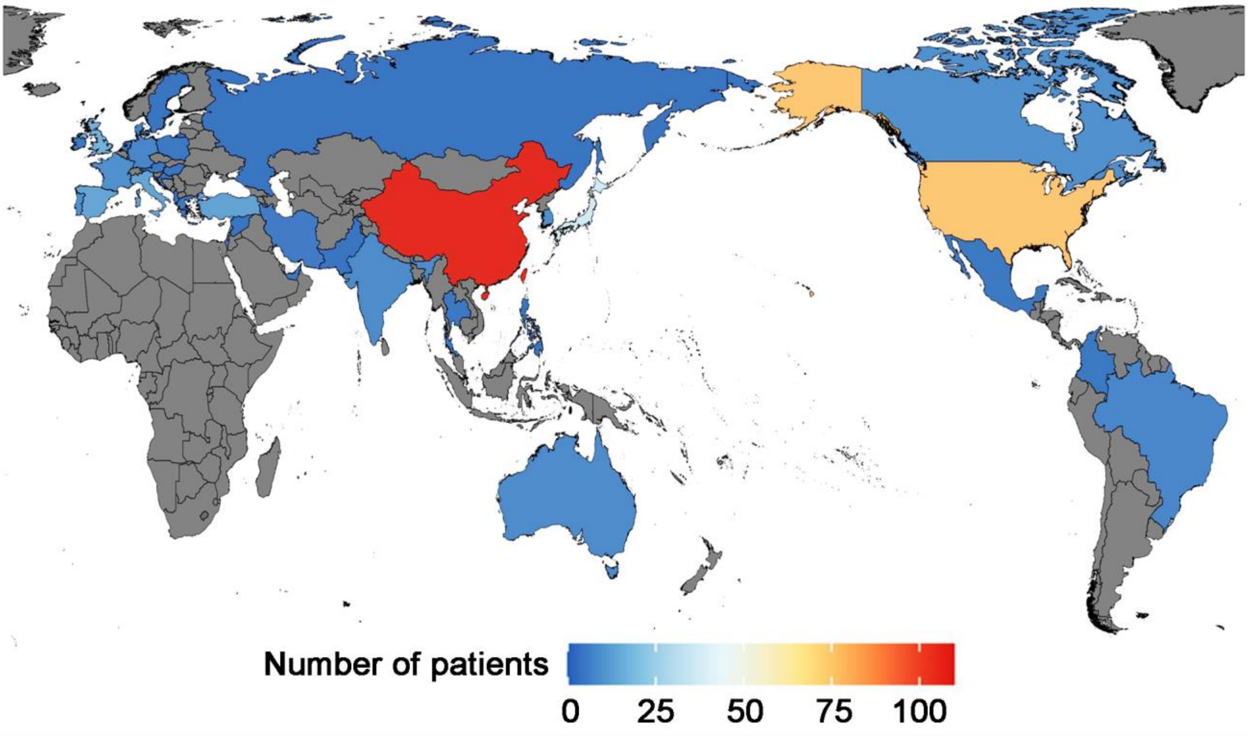

Figure S1. The global regional distribution of enrolled patients.

Table S1. Clinical characteristics of 38 IVL patients from Qilu Hospital of Shandong University

Cases	Age (years)	Gravidity/ parity	Clinical symptom	Clinical stage	History of surgery	Involved vessel	Length (cm)	Surgical approach	Completed excision	Ovarian preservation	Follow-up (months)	Recurrence
1	40	1/1	Abdominal distension	I	Cesarean section	Ovarian vein	17	Abdominal hysterectomy + bilateral salpingo-oophorectomy	Yes	No	177.5	No
2	35	2/1	Abdominal distension, lower limb swelling, dyspnea/dysuria	III	Transabdominal resection of myoma, abdominal hysterectomy	Cardiovascular	>20	Two-stage surgery	Yes	No	163.4	No
3	47	2/2	Abdominal pain/dyspnea	III	Transabdominal resection of myoma	Inferior vena cava	Unknown	Transabdominal resection of myoma	No	Yes	72.2	No
4	44	3/1	Abdominal discomfort	I	None	Ovarian vein	3	Abdominal hysterectomy + bilateral salpingo-oophorectomy	Yes	No	92.4	No
5	43	2/2	Abdominal pain	III	Abdominal hysterectomy	Inferior vena cava	>20	Abdominal hysterectomy + bilateral salpingo-oophorectomy	Yes	No	82.5	No
6	44	4/1	Abdominal distension, menorrhagia/dyspnea	II	Right salpingo-oophorectomy	Common iliac vein	6	Abdominal hysterectomy + left salpingo-oophorectomy	Yes	No	72.1	No
7	34	3/1	Abdominal distension	I	None	Ovarian vein	2.3	Transabdominal resection of myoma	Yes	Yes	28.4	Yes
8	36	3/1	Abdominal discomfort	III	Transabdominal resection of myoma	Cardiovascular	25	One-stage surgery	Yes	No	70.5	No
9	41	1/1	None	I	None	Ovarian vein	7	Abdominal hysterectomy + bilateral salpingo-oophorectomy	Yes	No	56.7	No
10	46	2/1	Dysuria	II	Abdominal hysterectomy	Common iliac vein	9.5	Abdominal hysterectomy + bilateral salpingo-oophorectomy	Yes	No	55.3	No
11	43	3/3	None	I	None	Uterine vein	Unknown	Laparoscopic hysterectomy + bilateral salpingectomy	Yes	Yes	36.1	Yes
12	47	3/3	Dysuria, lower limb swelling	III	Laparoscopic hysterectomy + bilateral salpingectomy	Inferior vena cava	12	Transabdominal resection of myoma + bilateral	Yes	No	51.8	No

								salpingo-oophorectomy				
13	45	2/1	Dizziness	IV	None	Pulmonary arteries	9	Abdominal hysterectomy + bilateral salpingo-oophorectomy	No	No	16.1	Yes
14	50	7/1	None	I	None	Uterine vein	<5	Abdominal hysterectomy + bilateral salpingo-oophorectomy	Yes	No	41.2	No
15	33	2/1	Menorrhagia, abdominal distension	I	Appendectomy	Uterine vein	3	Transabdominal resection of myoma	Yes	No	34.4	No
16	47	4/1	None	I	None	Uterine vein	2.5	Laparoscopic hysterectomy + bilateral salpingectomy	Yes	Yes	40.6	No
17	39	1/1	None	I	None	Uterine vein	<5	Laparoscopic hysterectomy + bilateral salpingectomy	Yes	Yes	27.8	No
18	39	1/1	None	I	None	Ovarian vein	<5	Transabdominal resection of myoma	Yes	Yes	40.5	No
19	41	4/3	None	II	None	Inferior vena cava	7.5	Abdominal hysterectomy + bilateral salpingo-oophorectomy	Yes	No	36.5	No
20	43	2/2	Dizziness	III	None	Cardiovascular	20	Two-stage surgery	Yes	No	34.9	No
21	66	2/2	Chest tightness	IV	None	Cardiovascular, pulmonary vascular	12	Intraoperative death	No	Yes	0	Death
22	48	2/1	Menorrhagia	II	None	Internal iliac vein	1.2	Laparoscopic hysterectomy + bilateral salpingo-oophorectomy	Yes	No	32.1	No
23	43	3/2	Abdominal pain	I	Cesarean section	Uterine artery	2.3	Laparoscopic hysterectomy + bilateral salpingectomy	Yes	Yes	32.9	No
24	33	1/1	None	I	Cesarean section	Uterine vein	1.5	Transabdominal resection of myoma	Yes	Yes	30.8	No
25	47	2/1	Menorrhagia	I	None	Uterine artery	3.3	Laparoscopic hysterectomy + bilateral salpingectomy	Yes	Yes	22.4	No
26	57	2/2	Abdominal distension	III	Abdominal hysterectomy	Cardiovascular	>20	One-stage surgery	Yes	No	18.6	No
27	48	3/2	Abdominal pain	I	None	Uterine vein	0.7	Abdominal hysterectomy + bilateral	Yes	No	15.3	No

								salpingo-oophorectomy				
28	48	8/2	None	II	None	Common iliac vein	10	Abdominal hysterectomy + bilateral salpingo-oophorectomy	Yes	No	14.6	No
29	35	3/1	None	I	Cesarean section	Ovarian vein	Unknown	Transabdominal resection of myoma	Yes	Yes	27.5	Yes
30	38	3/1	None	II	Transabdominal resection of myoma	Common iliac vein	3.5	Abdominal hysterectomy + right salpingo-oophorectomy	Yes	Yes	11.7	No
31	43	2/1	None	II	None	Internal iliac vein	>20	Abdominal hysterectomy + bilateral salpingectomy	Yes	Yes	20.2	No
32	58	6/3	Abdominal pain	II	Abdominal hysterectomy + left salpingo-oophorectomy	Common iliac vein	6	Right salpingo-oophorectomy	Yes	No	9.2	No
33	43	5/1	None	I	None	Ovarian vein	<5	Abdominal hysterectomy + left salpingo-oophorectomy	Yes	Yes	73.4	Yes
34	48	5/1	Abdominal pain	II	Abdominal hysterectomy + left salpingo-oophorectomy	Inferior vena cava	7.5	Right salpingo-oophorectomy	Yes	No	7.5	No
35	42	6/1	Urinary frequency	I	Laparoscopic myomectomy	Uterine vein	<5	Laparoscopic myomectomy	Yes	Yes	27.2	Yes
36	44	6/1	None	I	Laparoscopic myomectomy	Ovarian vein	<5	Laparoscopic hysterectomy + left salpingo-oophorectomy	Yes	Yes	5.2	No
37	47	1/1	Menorrhagia	I	None	Uterine vein	6	Laparoscopic hysterectomy + bilateral salpingectomy	Yes	Yes	4.8	No
38	37	4/1	None	I	Cesarean section	Uterine vein	7	Abdominal hysterectomy + bilateral salpingectomy	Yes	Yes	4.5	No

IVL, intravenous leiomyomatosis.

Table S2. Clinical characteristics of 11 deaths IVL patients from literature

Cases	Reference	Age (years)	Clinical stage	Surgical approach	Follow-up (months)	Recurrence	Causes of death
1	18	66	III	Abdominal hysterectomy + bilateral salpingo-oophorectomy	18	Yes	Severe heart failure
2	18	27	I	Subtotal abdominal hysterectomy + left salpingo-oophorectomy	12	Yes	Intraoperative massive retroperitoneal hemorrhage after recurrence
3	32	44	III	Abdominal hysterectomy + bilateral salpingo-oophorectomy	0.1	No	Postoperative retroperitoneal and pelvic hemorrhage
4	50	72	I	Transabdominal resection of myoma	24	Yes	Budd-Chiari syndrome
5	62	41	III	One-stage surgery	24	Yes	Diffuse abdominal metastases
6	63	47	III	Two-stage surgery	24	Yes	Died during atrial dissection
7	67	57	III	One-stage surgery	0	No	Intraoperative inferior vena cava laceration
8	95	47	IV	None	0	No	Cardiac arrest
9	194	64	Unknown	Abdominal hysterectomy + bilateral salpingo-oophorectomy	50	No	Died of unrelated cause
10	236	47	III	Tumor resection surgery	132	Yes	Multiple organ failure due to tumor progression
11	239	50	IV	None	0	No	Sudden right heart failure

IVL, intravenous leiomyomatosis
